# Kefir and Intestinal Microbiota Modulation: Implications in Human Health

**DOI:** 10.3389/fnut.2021.638740

**Published:** 2021-02-22

**Authors:** Maria do Carmo Gouveia Peluzio, Mariana de Moura e Dias, J. Alfredo Martinez, Fermín I. Milagro

**Affiliations:** ^1^Department of Nutrition and Health, Universidade Federal de Viçosa, Viçosa, Brazil; ^2^Department of Nutrition, Food Science and Physiology, Center for Nutrition Research, University of Navarra, Pamplona, Spain; ^3^Centro de Investigación Biomédica en Red de la Fisiopatología de la Obesidad y Nutrición (CIBERobn), Carlos III Health Institute, Madrid, Spain; ^4^Instituto de Investigación Sanitaria de Navarra, Navarra Institute for Health Research, Pamplona, Spain; ^5^Madrid Institute of Advanced Studies (IMDEA Food), Food Institute, Madrid, Spain

**Keywords:** kefir, probiotics, health, fermented foods, gut microbiota

## Abstract

In the last decades changes in the pattern of health and disease in Latin America and in the world has been observed, with an increase in cases of chronic non-communicable diseases. Changes in intestinal microbiota composition can contribute to the development of these diseases and be useful in their management. In this context, the consumption of fermented foods with probiotic properties, such as kefir, stands out due to its gut microbiota-modulating capacity. There is an increasing interest in the commercial use of kefir since it can be marketed as a natural beverage containing health-promoting bacteria and has been gaining international popularity in Latin America. Also the consumption of these drinks in Latin America seems to be even more relevant, given the socioeconomic situation of this population, which highlights the need for disease prevention at the expense of its treatment. In this narrative review, we discuss how kefir may work against obesity, diabetes *mellitus*, liver disease, cardiovascular disorders, immunity, and neurological disorders. Peptides, bioactive compounds and strains occurring in kefir, can modulate gut microbiota composition, low-grade inflammation and intestinal permeability, which consequently may generate health benefits. Kefir can also impact on the regulation of organism homeostasis, with a direct effect on the gut-brain axis, being a possible strategy for the prevention of metabolic diseases. Further studies are needed to standardize these bioactive compounds and better elucidate the mechanisms linking kefir and intestinal microbiota modulation. However, due to the benefits reported, low cost and ease of preparation, kefir seems to be a promising approach to prevent and manage microbiota-related diseases in Latin America and the rest of the world.

## Introduction

In the last decades, a major change in global health has been observed (1), with changes in the intestinal microbiota. The formation of intestinal microbiota during childhood is believed to be a key factor in the development of human diseases, such as allergies, neurological disorders and obesity (2–4), which indicates that the intestinal microbiota has a crucial role in the progress of new strategies for maintaining health.

The intestinal microbiota is a set of microorganisms that inhabit the human intestinal tract, and is composed by archaea, bacteria, fungi, helminths, and others (5). In a healthy adult individual, it is believed to be composed of more than 10^14^ microorganisms (6). According to the World Gastroenterology Organization stomach and duodenum have very low number of microorganisms, mainly lactobacilli and streptococci. Jejunum and ileum have an intermediate amount (10^4^-10^7^ cells per gram) (7) while large intestine has the largest amount with more than 10^12^ cells per gram, especially anaerobic microorganisms (7, 8). This microbial diversity also suggests the importance of the role that the intestinal microbiota plays in human health, indicating that—unlike what was thought in the past—microorganisms are not necessarily negative or the cause of diseases. On the contrary, it is increasingly clear that they co-evolved with human hosts and the presence of microorganisms can be important for maintaining health (1–4).

Knowledge on the importance of bacteria for human health dates back over 150 years (9), and fermented foods are important in this context. Through fermentation, food has contributed to the evolution of humanity due to its increased conservation capacity and shelf life, development of flavors, and health benefits (10, 11).

Able to resist to the digestive system, beneficial microorganisms occurring in fermented foods are able to act in the intestine. Fermented foods are able to modify the composition of the intestinal microbiota, improve the control of intestinal permeability, increase its barrier function (10–12), activate digestive enzymes, and assist in the production of short-chain fatty acids and vitamins. In addition, fermented foods have bioactive compounds and peptides with prebiotic, antimicrobial, anti-inflammatory, and anti-oxidant activities. Thus, the consumption of fermented foods has been reported to reduce the risk of incidence of certain diseases, such as metabolic syndrome, cardiovascular diseases, diabetes, and cancer; relieves lactose intolerance symptoms; and increases immunity and health in general (10, 11).

Probiotics stand out among fermented foods (10). According to the definition, probiotics are living microorganisms that provide health benefits to those who consume them (13, 14). From the beginning of Elie Metchnikoff's studies (1907) to the present day, *Lactobacillus* and *Bifidobacteria* are important species of lactic acid bacteria used as probiotic with evidence of their importance in human health (9, 10). In addition, in the scenario of diseases caused by changes in the intestinal microbiota, the use of probiotics stands out since the probiotics are capable of promoting homeostasis of the intestinal microbiota through mucin production, competition for pathogen adherence, inflammation control, pH change, cytokine production, as well as having immunomodulatory and anti-inflammatory properties, which results in a better healthy condition (7, 15).

Kefir is a fermented product (11, 16) formed by a single culture composed of lactic and acetic acid bacteria and yeast (17). It is a low-cost food, accessible to the general population, easy to handle and with great functional potential (18) due to its bioactive compounds (11, 18) like exopolysaccharides, conjugated fatty acids and peptidases (11, 16). Kefir consumption does not seem to result in negative effects (18) for adult humans (19) or animals (20). In kefir produced with whole milk, high cholesterol content can be observed, and in individuals with intolerance to lacteal proteins, allergic reactions may be observed, that can be avoided by replacing the milk matrices for water and sugar, which reinforces the absence of adverse effects of kefir (21).

Kefir peptides are able to improve parameters related to obesity in Sprague Dawley rats with diet-induced obesity. They act to inhibit lipogenesis by downregulating the fatty acid synthase (FAS) enzyme and increasing the phosphorylated acetyl coenzyme A carboxylase (p-ACC) protein; rising lipid oxidation and the expression of AMP-activated protein kinase (p-AMPK), peroxisome proliferator-activated receptor alpha (PPAR-α) and carnitine palmitoyltransferase 1 (CPT1); and decreasing the inflammatory response and oxidative modulation—with a reduction in tumor necrosis factor alpha (TNF-α), interleukin-1 beta (IL-1β) and transforming growth factor beta (TGF-β) cytokines (22) (Table 1). In addition, the strains found in kefir, such as *Lactobacillus harbinensis, Lactobacillus paracasei*, and *Lactobacillus plantarum*, have a role in the tolerance to bile acids and salts, in the adhesion of the intestinal mucosa and in antimicrobial resistance, indicating that bacteria occurring in kefir are capable of standing the gastrointestinal tract, as well as having probiotic and antioxidant activities (43).

**Table 1:** Main results of kefir intervention studies.

**References**	**Population**	**Disease/condition**	**Kefir consumption**	**Main outcomes in kefir groups**
**Human studies**				
Bellikci-Koyu et al. (19)	- Humans	- Metabolic syndrome	- 180 mL kefir/day; - 12 weeks of intervention time;	- ↑ Actinobacteria, fasting insulin and HOMA-IR; - ↓ pro-inflammatory cytokines, such as TNF-α and IFN-γ, and in systolic and diastolic pressure.
Fathi et al. (23)	- Women aged 25–45 years	- Obesity	- 2 servings/day; - 8 weeks of intervention time.	- ↓ serum levels/ratios of lipoproteins (TC, LDL, Non-HDL, TC/HDL, and LDL/HDL).
Ostadrahimi et al. (24)	- Humans aged 35–65 years	- Type 2 diabetes mellitus	- 600 mL/day of probiotic fermented milk containing *Lactobacillus casei, Lactobacillus acidophilus* and *Bifidobacteria*; - 8 weeks of intervention times.	- ↓ HbA1C
Ozcan et al. (25)	- Post-menopausal women	- Sleep quality, quality of life and depression	- 500 mL kefir daily (instructed to drink 250 mL in the morning and in the evening); - 30 days of intervention time.	- MENQOL, BDI, and WHIIRS scoresshowed significant changes
Praznikar et al. (26)	- Humans	- Obesity/intestinal integrity	- 300 mL of kefir/day; - 3 weeks of intervention time.	- ↓ serum zonulin levels, glucose and HDL cholesterol, and self-reported appetite perceptions; - ↑ the positive affect or mood.
St-Onge et al. (27)	- Humans (men)	- Hypercholesterolemy	- 500 mL kefir/day; - 2 periods of 4 weeks of intervention time.	- ↑ fecal isobutyric, isovaleric and propionic, the total amount of fecal short chain fatty acids, and fecal bacterial content.
**Animal studies**				
Bourrie et al. (28)	- C57BL/6 female mice	- Obesity induced by diet	- 100 μL kefir by gavage/day; - 12 weeks of intervention time.	- ↓ weight gain, plasma cholesterol, and hepatic triglyceride deposit.
Chen et al. (29)	- Sprague Dawley rats	- Hepatic steatosis induced by high-fat diet	- 10^7^ to 10^10^ CFU of *Lactobacillus mali* APS1 by gavage/day; - 12 weeks of intervention time.	- ↓ HOMA index, hepatic lipid accumulation; - ↑ GLP-1, hepatic antioxidant activity, butyrate and Bacteroidetes/Firmicutes.
Choi et al. (30)	- C57BL/6J mice	- Obesity induced by high-fat diet	- 0.1 to 0.2% kefir powder-supplemented high-fat diet - 8 weeks of intervention time.	- ↓ body weight, epididymal fat pad weight, adipocyte diameters, genes related to adipogenesis and lipogenesis, proinflammatory marker levels in epididymal fat, hepatic triacylglycerol concentrations, serum alanine transaminase, aspartate transaminase activities, serum triacylglycerol, total cholesterol, low-density lipoprotein-cholesterol.
Golli-Bennour (31)	- Wistar rats	- Hepatotoxicity	- Not informed	- Normalized the elevated serum levels of AST, ALT, total bilirubin, and cholesterol;
Kim et al. (32)	- Female BALB/c mice	- Obesity	- 0.2 mL kefir milk orally/day; - 3 weeks of intervention time.	- ↓ Firmicutes, Proteobacteria, Enterobacteriaceae and Firmicutes/Bacteroidetesratio; - ↑ Bacteroidetes, Lactobacillus, and Lactococcus, and total yeast; - Suppressed proliferation of the opportunisticpathogen Enterobacteriaceae.
Kim et al. (33)	- Male C57BL/6 mice	- Obesity and non-alcoholic fatty liver disease induced by 60% high-fat diet	- 0.2 mL of saline with 2 × 10^8^ CFU of *L. kefiri* DH5; - 0.2 mL of saline with 2 × 10^8^ CFU of *Leuconostoc. mesenteroides* DH4 - 0.2 mL of saline with 2 × 10^8^ CFU of *L. kefiri* DH7 - 6 weeks of intervention time.	-↓ body weight, epididymal adipose tissue weight, blood triglyceride, LDL-cholesterol levels, hepatic steatosis, adipocyte diameter; - Modulated gut microbiota; - Upregulated PPARα, FABP4, and CPT1 expression in the epididymal adipose tissues
Kim et al. (20)	- Dogs	- Quality of life	- 200 mL of kefir once a day (*ad libitum*); - 2 weeks of intervention time.	- ↑ lactic acid bacteria and lactic acid bacteria: Enterobacteriaceae ratio; - ↓ Firmicutes: Bacteroidetes ratio.
Le Barz et al. (34)	- C57BL/6 mice	- Obesity induced by diet	- 1 of 3 Lactobacillus strains (Lb38, *L. plantarum*; L79, *L. paracasei/casei*; Lb102, L. *rhamnosus*) or *Bifidobacterium* strains (Bf26, Bf141, 2 different strains of B. *animalis* ssp. *lactis*) administered with diet at 10^9^ CFU/day; - 8 weeks of intervention time.	- ↓ diet-induced obesity, visceral fat and inflammation; - ↑ glucose tolerance, insulin sensitivity and intestinal integrity.
Lim et al. (35)	- C57BL/6J mice	- Obesity induced by high-fat diet	- 5% water-soluble EPS and 8% resides after EPS removal from the probiotic kefir in diet; - 4 weeks of intervention time.	- ↓ body weight gain, adipose tissue and plasma very low-density lipoprotein cholesterol; - ↑*Akkermansia spp*. in feces.
Noori et al. (36)	- Rats	- Nicotine cessation-induced anxiety, depression and cognition impairment	- 5 mg/kg/day of cow milk kefir or soy milk kefir; - 7 days of intervention time.	- Improved anxiety, learning and memory impairment; - ↓ the severity of depression.
Rosa et al. (37)	- Spontaneously Hypertensive Rats	- Metabolic syndrome	- 1 mL kefir/day; - 10 weeks of intervention time.	- ↓ plasma triglycerides, liver lipids, liver triglycerides, insulin resistance, fasting glucose, fasting insulin, thoracic circumference, abdominal circumference, products of lipid oxidation, pro-inflammatory cytokine expression (IL-1β); - ↑ anti-inflammatory cytokine expression (IL-10).
Sun et al. (38)	- Kunming male mice	- Depression	- *L. kefiranofaciens* ZW3 a dose of 10^7^ CFU, 10^8^ CFU and 109 CFU/mouse/day; - 6 weeks of intervention time.	- Improved depression-like behavior and independent exploration ability; - Regulated biochemical disorders in the hypothalamic–pituitary–adrenal axis, immune system and tryptophanmetabolism caused by stress; - Modulated the composition of the gut microbiota, and alleviateconstipation.
Tiss et al. (39)	- Wistar rats	- Obesity, type 2 diabetes, hyperlipidemia and liver-kidney toxicities in high-fat-high-fructose diet	- 10 mL/kg of body weight of a fermented soymilk by kefir; - 90 days of intervention time.	- ↓ pancreas lipase and alpha-amylase; - Reverted back all these histological toxicities.
Tung et al. (22)	- Sprague Dawley rats	- Obesity induced by high-fat diet	- 164 mg/kg of body weight of kefir peptides; - 8 weeks of intervention time.	- ↓ FAS enzyme, inflammatory response and oxidative modulation (TNF-α, IL-1β and TGF-β cytokines); - ↑ p-ACC protein, fatty acid oxidation and expression of p-AMPK, PPAR-α and CPT1.
Tung et al. (40)	- ApoE knockout mice	- High fat diet-induced atherosclerosis	- 100 mg/kg low-dose kefir peptides powder; - 400 mg/kg high-dose kefir peptides powder; - 12 weeks of intervention time.	- Improved atherosclerotic lesion development by protecting against endothelial dysfunction; - ↓ oxidative stress, aortic lipid deposition, inflammatory immune response, fibrosis; - Attenuating macrophageaccumulation.
Vinderola et al. (41)	- BALB/c mice	- Immunomodulation	- 3.1 ± 0.3 ml/kefir daily; - 2, 5, or 7 consecutive days of intervention time.	- Modulated the mucosal immune system in a dose-dependent manner
Wouw et al. (42)	- C57BL/6J mice	- Brain physiology and behavior	- 0.2 mL/kefir daily; - 3 weeks of intervention time.	- Ameliorated the stress-induced; - ↓ serotonergic signaling; - ↑ fear-dependent contextual memory; - Stimulate the production of GABA neurotransmitter.

*Where: ALT, alanine aminotransferase; AST, aspartate aminotransferase; BDI, beck depression inventory; CFU, colony forming unit; CPT 1, carnitine palmitoyltransferase 1; EPS, exopolysaccharides; FABP4, fatty acid-binding protein 4; FAS, fatty acid synthase enzyme; GABA, gamma aminobutyric acid; GLP-1, glucagon-like peptide; kg, kilogram; HbA1C, glycated hemoglobin; HDL, high-density lipoprotein cholesterol; HFD, high fat diet; HOMA-IR, insulin resistance index; IFN-γ, Interferon-gamma; IL-1β, interleukin-1 beta; IL-10, interleukin−10; IT, intervention time; LDL, low-density lipoprotein cholesterol; MENQOL, menopause-specific quality of life questionnaire; mg, milligram; mL, milliliters; p-AMPK, AMP-activated protein kinase; PPAR α, peroxisome proliferator-activated receptor alpha; TC, total cholesterol; TGF-β, transforming growth factor beta; TNF-α, tumor necrosis factor alpha; WHIIRS, the women's health insomnia rating scale; μL, microliters; ↑, increase; ↓, decrease*.

Given the above, we will discuss studies with animals and humans that reported the role of kefir and its bioactive compounds on the treatment of diseases characterized by shifts in intestinal microbiota.

## Kefir In Latin America

Non-communicable diseases are those diseases with a long duration and multifactorial causes which include genetic, psychological, environmental and behavioral factors. Also known as chronic diseases, some examples are cardiovascular disease, cancer and diabetes (44).

The consumption of milk-based fermented drinks is related to the prevention of chronic non-communicable diseases, with an association between the consumption of yogurt and a reduction in weight gain, and lower risk of obesity and cardiovascular diseases (45). Fermented milks also aid in the digestion of lactose and are a source of calcium and protein, therefore presenting interesting nutritional value (18, 46).

The consumption of yogurt and fermented drinks varies widely among countries: Brazil has a consumption similar to the United States (46) being, Argentina and Chile the countries with the highest consumption in Latin America (45).

According to data from the American Study of Nutrition and Health (ELANS), which aims to identify the consumption of food directly related to the occurrence of chronic non-communicable diseases, < 3.5% of the Latin American population has a good consumption of yogurt (45). This consumption is considered low and indicates that there is a consumption gap to be filled, since fermented drinks can contribute to improving the health of those who consume it (46), contributing to the prevention and treatment of chronic non-communicable diseases.

In Brazil, the consumption of yogurt has been increasing among populations with higher income, being typically consumed outside home (47) and, in Latin America, a similar movement is observed, with consumption mainly among the youngest and with highest socioeconomic level (45). Therefore, as an alternative to the consumption of yogurt, we highlight the use of milk kefir, which is also a fermented product, with health benefits due to its probiotic content and the presence of bioactive compounds (18).

Medicines and supplements for the treatment of chronic non-communicable diseases, such as probiotics, can have significant costs for the Latin American population, which may turn their consumption impossible (48). Kefir, on the other hand, can be produced by the consumer himself, using baits acquired by donation, which significantly reduces production and consumption costs (18, 49–51). This allows the use of kefir by a larger portion of the Latin American population. In addition, among the high-income population, kefir consumption also stands out, as there is a tendency, in this audience, to seek healthier and more nutritious foods, such as kefir (52).

The benefits of kefir have not yet been widely publicized among the Latin American population. However, scientific interest in this fermented product is gaining more researchers in Latin America. The antifungal property of kefir, for example, has been studied both in the production of cereals and in the preservation of food. In a study by Gamba et al. (53) there is significant resistance to natural and artificial fungal contamination in arepas (typical dish from Venezuela, Colombia, Bolivia, and Panama) cooked in kefir. In addition, the arepas maintained the organoleptic characteristics of the traditional product after adding kefir, with an improvement in the product's useful life.

The use of kefir to treat diseases has also been studied by Latin American researchers, observing reductions in pre-neoplastic lesions in the intestinal colon (54) and improvement of parameters related to obesity after consumption of this fermented drink (37) in animal models. Better quality of life for individuals with lactose intolerance and osteoporosis (55) has been also observed.

Studies analyzing the effects of kefir on the modulation of the intestinal microbiota (56) and the immune system (57) have also been carried out. In addition, the microbial non-kefir fraction, known as post-biotics (metabolites produced in the fermentation process of dietary components as well as the endogenous components generated by bacteria-host interactions that influence human health) have been widely studied due to their antagonism against pathogenic bacteria such as *Escherichia coli, Salmonella* spp. and *Bacillus cereus* (58, 59). This indicates that kefir produced in Latin America is indeed a food with health potential, being interesting its use by the Latin American population.

## Modulation of the Intestinal Microbiota

The components of intestinal microbiota, that is, archaea, fungi, helminths, bacteria and other microorganisms (5), can take two forms: in balance or not. In the first case, known as eubiosis, microbiota tolerates small changes, which may come from the environment, the diet or the water consumed, presenting flexibility to maintain its balance. Cases of major changes, however, such as translocation or growth of a specific bacterial groups, colonization by pathogenic bacteria, use of antibiotics, and changes in lifestyle, lead to imbalance, that is, dysbiosis (60, 61).

Regardless of balance, intestinal microbiota affects the functionality of various organs such as brain, liver, pancreas, intestine, and heart (60, 61). Furthermore, the intestinal microbiota participates in the development and maturation of organs and physiological processes (60), which suggests that modulation of gut microbiota may be a key event for the treatment of diseases and maintenance of health.

Fortunately, modulation of the intestinal microbiota is a reality. Through technological advances, such as interventions with bariatric surgery (62) or fecal transplant (63), or via food (19, 20, 32), the obtained results are promising, showing, in fact, changes in the composition of the intestinal microbiota.

In a longitudinal study that evaluated obese patients undergoing bariatric surgery, body weight reduction and metabolic improvements occurred via modulation of the intestinal microbiota. Compared to individuals of normal weight, patients who underwent bariatric surgery had variation in the *Firmicutes, Fusobacteria*, and *Verrucomicrobia* phyla. In addition, after bariatric surgery, the presence of *Akkermansia muciniphila* was observed. It is a species positively associated with lipid metabolism and negatively associated with adipose tissue inflammation and with circulating levels of glucose, insulin, leptin, and triglycerides, which suggests that its presence indicates improvement in the expression of healthy metabolism markers (62).

Fecal microbiota transplantation also shows its potential modulating role. In a double-blind study with 22 obese patients [body mass index (BMI) ≥ 35 kg/m^2^] without associated metabolic changes, such as type 2 diabetes, non-alcoholic hepatitis, and metabolic syndrome, the researches worked by offering the transplantation of fecal microbiota (TMF) of a thin donor using capsules (63).

The consumption of TMF capsules was considered safe. A positive modulation of the intestinal microbiota was observed. At the beginning of the experiment, obese individuals had an intestinal microbiota with a specific profile; however, after TMF intake, it was observed a similarity between the microbiota profile of obese patients and that of the thin donor, as well as changes in beta diversity, that is, changes in the species composition of the microbiota (63).

Reduction of taurocholic acid was also observed in the feces of individuals who received TMF (63), and this change is related to the alterations seen in the composition of the intestinal microbiota. Secondary bile acids, for example, are produced in the colon and are influenced by the microbiota present on it. This production varies according to the colonic microbiota and may contribute to a state of health or disease (64).

In addition, bile acids have antibacterial capacity. They also participate in the progression of diseases associated with changes in intestinal microbiota, such as obesity and gastrointestinal diseases, contributing to the modulation of inflammatory processes and signaling energy metabolism events and through their performance as a biological detergent (64). Hence, there is a two-way path where both gut microbiota influences the production of bile acids, and bile acids contribute to intestinal modulation performance.

In a study that sought to evaluate the role of kefir in the modulation of the intestinal microbiota of mice, it was observed that the consumption of this drink was not able to change the total number of bacteria in intestinal microbiota; however, when evaluating the phyla in the group that consumed kefir, a reduction in Enterobacteriaceae and an increase in *Lactobacillus* and *Lactococcus* contents were observed during the 3-week intervention period; also, a decrease in Firmicutes and Proteobacteria and an increase in Bacteroidetes, *Lactobacillus* and *Lactococcus* at the end of the experiment was observed. On the other hand, there was a significant increase in fecal yeasts after the consumption of kefir (32) (Table 1).

Such results indicate that kefir was able to improve the intestinal microbiota of mice that consumed it, with an emphasis on Enterobacteriaceae reduction. This is considered a pathogenic family, usually dysregulated in situations of behavioral and metabolic changes—such as the consumption of diets which are high in fat and low in fiber, during aging, and in cases of inflammation—(32) emphasizing that such eating habits and inflammation are characteristic of obesity (62, 65–67).

Similarly, a study with dogs confirmed that kefir was able to modulate intestinal microbiota. It decreased the Firmicutes:Bacteroidetes ratio and increased the lactic acid bacteria:Enterobacteriaceae ratio, which suggests an improvement in the animal's health. There were also changes in phylum, family and species level, which indicates that kefir was able to modulate the canine intestinal microbiota (20) (Table 1).

In humans, modulation of intestinal microbiota also occurs after the consumption of kefir, as discussed by Bellikci-Koyu et al. (19) who investigated individuals with metabolic syndrome that were supplemented with kefir for 12 weeks (Table 1). After the intervention, there was a significant increase in *Actinobacteria*, as well as changes in the genera of the phyla Bacteriodetes and Firmicutes, in the group that consumed kefir (19).

This intestinal modulation interferes with metabolic parameters, characteristic of metabolic syndrome, showing improvement in fasting insulin and insulin resistance index (HOMA-IR), and a decrease in pro-inflammatory cytokines, such as TNF-α and interferon-gama (IFN-γ), and in systolic and diastolic pressure. Moreover, a correlation was observed between these parameters and the intestinal microbiota: body weight gain and BMI were positively correlated with the relative abundance of Firmicutes and Proteobacteria and negatively correlated with the relative abundance of Clostridia. Correlations between the composition of the intestinal microbiota and the amount of fat mass, waist circumference, LDL-cholesterol, homocysteine, insulin, and blood pressure were also found (19).

There are many factors that can alter the composition of the intestinal microbiota in humans, such as age, sex, initial formation of intestinal microbiota, food consumption—with emphasis on fiber consumption–, lifestyle, and use of medications—especially antibiotics (1–4). Therefore, the results observed by Bellikci-Koyu et al. (19) are relevant, paving the way for further studies that prove the modulation of the human intestinal microbiota through the use of kefir in individuals with metabolic syndrome.

## Kefir and Obesity

Dysbiosis and other determinants of intestinal microbiota formation in childhood, such as birth via cesarean section or formula feeding, are associated with a higher risk of developing obesity, both in child and adulthood (3, 68). Children treated with antibiotics, specially macrolides, amoxicillin, cefdinir, vancomycin, and tetracyclines (69) had a higher incidence of obesity (3), since antibiotics can alter the composition of the intestinal microbiota, leading to a dysbiotic state (3, 70). In addition, the intestinal microbiota is demonstrably different in obese and eutrophic conditions (62, 71), which reinforces the relationship between intestinal microbiota and obesity. Thus, obesity and overweight, characterized by the excess or accumulation of body fat with a consequent increase in health risk (72), can also be influenced by the intestinal microbiota.

Kefir could act against obesity by inhibiting enzymes related to the digestion of carbohydrates and lipids, which will result in less energy release. For example, Tiss et al. (39) using a fermented drink with kefir produced from soymilk, evaluated the activity of lipase and α-amylase *in vitro* and in the intestine and pancreas of rats under high-calorie diet-induced obesity (Table 1).

In the *in vitro* analysis, the study showed the ability of kefir to inhibit α-amylase and pancreatic lipase. Authors assumed that this ability is related to the presence of isoflavone aglycones, such as genistein, daidzein and glycitein, present in the drink after the fermentation process. In the *in vivo* section, the obese animals treated with the fermented drink were more stimulated to perform physical activity. Intestinal and pancreatic lipase activity decreased in the groups receiving kefir, leading to a reduction in total cholesterol and LDL-cholesterol, and an increase in HDL-cholesterol rates, as well as body weight loss. Inhibition of intestinal and pancreatic α-amylase activity and, consequently, decreased blood glucose and protection of liver and kidney tissues from toxicity were also observed after kefir consumption; that is, this fermented drink was able to reverse parameters related to obesity (39).

Bourrie et al. (28) also evaluated kefir regarding the reduction of weight gain and plasma cholesterol in C57BL/6 female mice with obesity induced with a diet of 40% calories from fat and 1.25% cholesterol. The animals received 100 μL of kefir or milk over a period of 12 weeks, with 4 different drinks of traditional kefir and 1 of commercial kefir (28) (Table 1).

Among traditional kefir, two types decreased weight gain and plasma cholesterol and one type reduced hepatic triglyceride deposit, which indicates its potential in controlling obesity with improved metabolic function. The difference between the results is due to the different microbiological compositions, viscosity, and pH of fermented drinks (28).

Kim et al. (33) evaluated the anti-obesity effects of kefir in C57BL/6 mice with high-fat diet-induced obesity and non-alcoholic fatty liver disease (Table 1). The *Lactobacillus kefiri* DH5 strain was able to decrease body weight, adipose tissue and plasma lipid parameters, acting through the reduction of cholesterol in the intestinal lumen and the upregulation of PPARα in adipose tissue. PPARα is a transcription factor involved in the process of lipid oxidation and consequent metabolism of carbohydrates and lipids, noting that its activation is related to an increase in hepatic steatosis and inflammation (73).

Moreover, the animals that consumed this strain presented a variation in the composition of their intestinal microbiota, with a lower number of Proteobacteria and Enterobacteriaceae, when compared to the non-supplemented animals. These results indicate that *Lactobacillus kefiri* DH5 is a potential probiotic strain for the treatment of obesity (33).

Lim et al. (35) evaluated the effect of exopolysaccharides derived from kefir grains, showing that the beneficial effects found could be related to the viscosity of exopolysaccharides produced by the bacteria present in kefir (Table 1). Authors observed that exopolysaccharides were able to suppress obesity *in vitro*, through the supply of adipogenesis. Also, reductions in body weight gain, adipose tissue weight and plasma very low-density lipoprotein cholesterol concentration (VLDL) occurred *in vivo* (35).

Such *in vivo* results were explained by both the presence of bacterial metabolites and by the product's viscosity, which generates appetite suppression and reduces energy consumption as well as glucose and lipid absorption. Furthermore, the supply of kefir exopolysaccharides was able to increase the abundance of *Akkermansia* (35). *Akkermansia muciniphila* undergoes changes according to the diet consumed, modulates the intestinal microbiota, changes inflammatory conditions in adipose tissue, and improves metabolic parameters, like body weight, adiposity, inflammation markers and biochemical parameters, which suggests a great potential for the treatment of obesity (74).

## Kefir and Diabetes Mellitus

The development of diabetes mellitus is associated with low-grade chronic inflammation. Changes in intestinal permeability, which are favored by an imbalance in the intestinal microbiota, encourage the occurrence of this inflammation, which leads to resistance to systemic insulin, with consequent development of diabetes (3, 75). In addition, factors that affect the formation of the intestinal microbiota—such as maternal health during pregnancy, birth via cesarean section, the use of antibiotics during childhood, and the presence of intestinal dysbiosis in childhood—are also related to the development of diabetes (3).

In a study with Wistar rats with monosodium glutamate-induced metabolic syndrome, it was observed that whole milk kefir (via gavage, for 10 weeks) was able to reduce insulin resistance. Such results were attributed to the calcium content consumed by the animals, as well as the bioactive compounds produced during the fermentation of kefir. Moreover, kefir stimulated uptake of glucose by muscle cells, which contributed to the reduction of insulin resistance (37) (Table 1).

*Lactobacillus mali* APS1 is a strain isolated from kefir grain that may be useful in the treatment of diabetes. In a study (Table 1) with mice consuming a high-fat diet, the administration of this strain was able to reduce serum glucose and HOMA index, increasing the levels of glucagon-like peptide (GLP-1) and butyrate (29). The reduction in the HOMA index indicates glycemic control (76) and the increase in GLP-1 indicates control of hunger and possible protection of pancreatic beta cells, which are insulin-producing cells, essential for maintaining glycidic homeostasis (77). In addition, a recent review discusses the decrease in butyrate content as a characteristic of intestinal dysbiosis in diabetes (78). Therefore, these results are positive.

In humans, a beneficial role of kefir in the treatment of diabetes mellitus has been also observed, as discussed in a work with 60 diabetic patients, aged 35–65 years. The patients were divided into 2 groups: the kefir probiotic group and the conventional fermented milk group, and both received 600 mL/day of the treatment drink for 8 weeks. After the intervention, patients supplemented with kefir presented lower values of fasting glucose and glycated hemoglobin than those that received the other fermented drink (24) (Table 1).

The healthy outcomes generated by kefir were attributed to its probiotic composition, mainly *Lactobacillus* and *Bifidobacterium*. These bacteria present a hypoglycemic effect since they stimulate the production of insulinotropic peptides and glucagon-like peptides, leading to an increase in the uptake of glucose by muscle cells, as well as stimulating the production of hepatic glycogen, which uses the glucose available in the bloodstream (24).

## Kefir and Liver Diseases

Toxins produced by intestinal microbiota and a picture of metabolic endotoxemia—that is, altered intestinal permeability—allow the development of a low-grade chronic inflammation. This inflammatory condition stimulates the activation of toll-like receptors and macrophages, which generates hepatic and systemic inflammation, explaining the relationship between the intestinal microbiota and the occurrence of liver diseases (75).

In addition to its effect on obesity, the consumption of *Lactobacillus kefiri* DH5 presented a hepatoprotective effect. The visual aspect of the liver of the animals that consumed this strain was similar to the animals that did not consume a high-fat diet, as well as presenting, microscopically, less lipid accumulation and smaller fat cells (33).

The hepatoprotective capacity of kefir was also assessed by Golli-Bennour et al. (31) who studied the effect of kefir on hepatotoxicity caused by a pesticide: deltamethrine (Table 1). In Wistar rats, the authors observed that deltamethrine altered liver parameters, such as aspartate aminotransferase (AST), alanine aminotransferase (ALT), bilirubin, and cholesterol, when compared to control groups without the pesticide; however, with kefir intake, these parameters were lower. Furthermore, the supply of pesticide and kefir was able to decrease the levels of carbonylated protein and malondialdehyde, as well as increasing the levels of catalase and superoxide dismutase when compared to the group that received only deltamethrine. These protein and lipid peroxidation disorders indicate oxidative stress and toxicity development by the pesticide. On the other hand, kefir was not able to induce oxidative stress by itself, and was able to revert the inflammatory condition (31).

Less histological and DNA damage was also observed in groups treated with pesticide and kefir when compared to the groups that received only deltamethrine, with this histological and genetic disarchitecture resulting from the inflammatory process previously described. It was concluded that kefir was not only able to revert the inflammatory condition but did not generate any damage, since the group treated with kefir alone obtained similar results to the control group. This finding indicates that kefir has a good antioxidant capacity (31), being a potential tool in the prevention and treatment of liver damage caused or mediated by oxidative stress.

The effect of *Lactobacillus mali* APS1, isolated from sugary kefir grains, was also tested in the fatty liver of rat fed with a high-fat diet, showing a significant reduction in weight, weight gain, hepatic lipid accumulation, and serum levels of AST and ALT. This strain acted through changes in intestinal microbiota composition, reducing the proportion of bacteria associated with non-alcoholic liver diseases and regulating lipid metabolism and oxidative stress response, which leads to suppression of hepatic steatosis progression (29).

## Kefir and Cardiovascular Changes

Cardiovascular diseases are also related to obesity's intestinal dysbiosis (4). In addition, changes in the intestinal microbiota can lead to the production of compounds such as trimethylamine N-oxide (TMAO), which increase the risk of developing cardiovascular diseases (79, 80).

Tung et al. (40) evaluated the effect of kefir peptides in Apo E –/– mice with high-fat diet-induced atherosclerosis (Table 1). After a 12-week intervention, the consumption of kefir led to a decrease in the evolution of atherosclerotic lesions, with less lipid deposition at the root of the aorta and suppression of the inflammatory immune response, through a reduction in oxidative stress, the accumulation of macrophages and the release of IL-1β and TNF-α cytokines. Moreover, kefir prevented the endothelial adhesion of monocytes, decreasing the evolution of the atherosclerotic lesion. These results indicate that the consumption of kefir could be useful in the prevention and treatment of atherosclerosis (40).

One of the risk factors for the occurrence of cardiovascular diseases is dyslipidemia. In this sense, in the host, there is less diversity of intestinal microbiota and a greater chance of dysbiosis. That is, a greater chance of inflammation and changes in intestinal permeability is observed, with negative consequences for the health of the host (81).

In dyslipidemia, changes in the production of short-chain fatty acids and bile acids have been observed. Short-chain fatty acids are metabolite substrates that participate in energy production, lipogenesis, gluconeogenesis, and cholesterol synthesis. On the other hand, primary bile acids can bind to the farnesoid X receptor, and this molecule is also related to the development of obesity. Both factors participate in lipid metabolism and intestinal microbiota change (81), indicating that modulation of intestinal microbiota may be a therapeutic alternative to prevent dyslipidemia.

Kefir may be an option in the treatment of dyslipidemia. Choi et al. (30) observed that kefir prevented the increase of lipid parameters in mice fed with an obesogenic diet, proposing that kefir acted by preventing lipid intestinal absorption (Table 1). In humans, the consumption of kefir drink (250 ml) for 8 weeks also improved the lipid profile, which was similar to the control group that consumed low-fat milk. This improvement in the lipid profile was related to the loss of body weight, achieved with the consumption of kefir, as well as to the changes generated in the intestinal microbiota, which led to an increase in the production of short-chain fatty acids and bile acids (23) (Table 1); however, it is noteworthy that the amount of kefir offered and the intervention time are fundamental to achieve the desired result for improving dyslipidemia (27) (Table 1).

## Kefir and Immunity

The correct formation of intestinal microbiota during childhood is fundamental for the complete development of the baby, since the intestinal microbiota is part of the immune system, being important especially in the first months of life, when the rest of the immune system is still forming. In this context, premature babies may have immaturity in the immune, respiratory and neurological systems, suggesting a possible relationship between them (3). In addition, the immune system and the intestinal microbiota play a symbiotic function, maintaining a picture of non-inflammatory homeostasis: in cases of intestinal dysbiosis, there is an activation of the immune system, which leads to changes in the host's immunity. These changes can impair the maturation of the innate immune system or lead to autoimmune diseases, such as type 1 diabetes (61).

The immunomodulatory capacity of kefir has been tested in BALB/c mice by using different concentrations of commercial kefir (diluted at 1/10, 1/50, 1/100, or 1/200 proportions) and pasteurized kefir (diluted at 1/6, 1/10, 1/50, 1/100 proportions). The results show that kefir modulates the immune response in a dose-dependent manner by stimulating intestinal IgA production and inhibiting the Th1-type immune response (41) (Table 1).

Le Barz et al. (34) also evaluated kefir for probiotic strains with immunometabolic properties (Table 1). *Lactobacillus rhamnosus* Lb102 and *Bifidobacterium* Bf141 showed good results in the treatment of obesity and in metabolic syndrome, with a reduction in visceral fat and inflammation, and an improvement in glucose tolerance and insulin sensitivity. These results were explained by a modulation of the intestinal microbiota and the maintenance of intestinal integrity by the probiotics (34).

In humans, the action of kefir on intestinal integrity has also been observed. A study encompassing 28 healthy, overweight and asymptomatic adults found that the use of kefir modified serum zonulin concentration (26) (Table 1). Zonulin is a protein that participates in the integrity of tight junctions, that is, the maintenance of intestinal integrity. In the presence of tight junctions, there is control of the passage of molecules through the paracellular content, which prevents the development of inflammatory processes and the occurrence of diseases. However, the increase in zonulin production leads to loss of function of the intestinal barrier with consequent passage of antigens through the paracellular content and activation of the innate immune response (82).

The probiotic capacity of kefir was able to modulate intestinal microbiota composition, which prevented excessive intestinal permeability by increasing serum zonulin concentrations. Consequently, there was a control of low grade chronic inflammation generated in cases of altered intestinal permeability, as has been proposed in obesity (26).

## Kefir and Neurological Changes

Neurological changes have multifactorial causes and intestinal microbiota also participates in this process. The composition of intestinal microbiota impacts the health of the microglia and the development of neuronal circuits, which contribute to neurological health (4). In addition, individuals with autism have a characteristic intestinal microbiota, which strengthens the relationship between intestinal microbiota and neurological disorders (1–4).

Noori et al. (36) assessed the role of kefir, fermented in both soy and cow's milk, in the treatment of depression, anxiety and cognitive impairment in an animal model subjected to stress due to the use of nicotine (Table 1). For this purpose, the animals were submitted to elevated plus maze (EPM) to assess anxiety, open field test (OFT) to assess locomotor activity and anxiety and forced swim test (FST) to assess depression. Both types of kefir were able to improve anxiety, decrease the severity of depression, and improve cognitive function throughout the treatment. Since kefir is a food rich in tryptophan, which is the precursor of serotonin, it is believed that kefir may be able to act on serotonin metabolism (36). Depression is related to changes in neuroplasticity being serotonin a neuromodulator capable of stimulating the development of neuronal plasticity (83). So, modulation in serotonin is a classic way of treating depression (36, 83).

Kefir has been reported to protect neurons from degradation through its anti-inflammatory capacity. Additionally, kefir may be able to activate receptors in the brain that stimulate learning and memory. So, in view of the cognitive improvement found, kefir can be potentially used for both prevention and treatment of depression and anxiety, especially in cases related to nicotine consumption. The authors believed that the results can be extrapolated to humans; however, further studies are needed to confirm this hypothesis (36).

The ability to modulate the intestinal microbiota and, consequently, anxiety and depression also suggests a positive role of kefir in these diseases (38, 42) (Table 1). Animals fed with kefir presented a specific intestinal microbiota composition, which presumably acts positively on the gut-brain axis. In addition, through analysis of the microbiome, authors suggest that kefir was able to stimulate the production of the gamma aminobutyric acid (GABA). It is hypothesized that *Lactobacillus reuteri* possibly converted 2-oxoglutarate to glutamate, which was subsequently converted into GABA, because of modulation of the intestinal microbiota by kefir, that leads to an increase in the production of *Lactobacillus reuteri*, a bacterial strain with benefits to the host's immune and metabolic system (42).

Kefir was also evaluated in an animal model simulating human depression, with stress induced through 7 stressors during 6 weeks. Mice supplemented with *Lactobacillus kefiranofaciens* ZW3 strain, isolated from kefir, had more movement (which indicates an increase in the ability to explore the environment and socialize), a greater preference for sucrose (which indicates that the animals returned to have pleasure) and a higher amount of water in their stools (which indicates a lower probability of having constipation, which is a condition associated with the occurrence of depression). In addition, there was an improvement in tryptophan metabolism, an increase in anti-inflammatory cytokines, a reduction in pro-inflammatory cytokines, and changes in the composition of the intestinal microbiota, with an increase in Actinobacteria, Bacteroides, Lachnospiraceae, Coriobacteriaceae, Bifidobacteriaceae *and Akkermansia*, and reduction in Proteobacteria (38).

All of these benefits indicate that the consumption of kefir was able to alter the metabolic pathways that led to the development of depression; however, further studies are needed to identify the optimal dosage to be consumed, so that the benefits of kefir are acquired by the host that consumes it (38).

The role of kefir on neurological diseases has also been studied in humans. For example, Ozcan et al. (25) assessed the consumption of kefir on sleep quality, quality of life and depression in post-menopausal women (Table 1). The consumption of kefir was positively correlated with the quality of life and quality of sleep, which might be a simple strategy, with good cost benefit and an alternative for the treatment of menopause; however, there were no promising results regarding to depression (25).

Studies that evaluate the psychobiotic activities of kefir on depression may be of great relevance, since the consumption of most antidepressants can generate side effects such as weight gain. In a study performed in Canada, for example, there was an association between obesity and high prevalence of antidepressants' prescription. Moreover, a greater prescription of antidepressants in the most severe grades of obesity (classes II and III) is commonly observed. The use of kefir, in this case, is particularly interesting since the use of medications can worsen obesity and decrease the chance of a positive response to treatment (84). Kefir could also be useful for the care of obese and depressed patients, although more intervention studies should be undertaken to properly evaluate its potential.

## Conclusions

The inclusion of kefir in the Latin American market presents a good alternative as an adjuvant therapy in non-communicable diseases and may display an economic prognosis, since, in 2016, the value of kefir market in Latin America was amounted to 150.8 million U.S. dollars and it is expected to rise to about 204.7 million U.S. dollars by 2021 (85).

**Figure 1:**
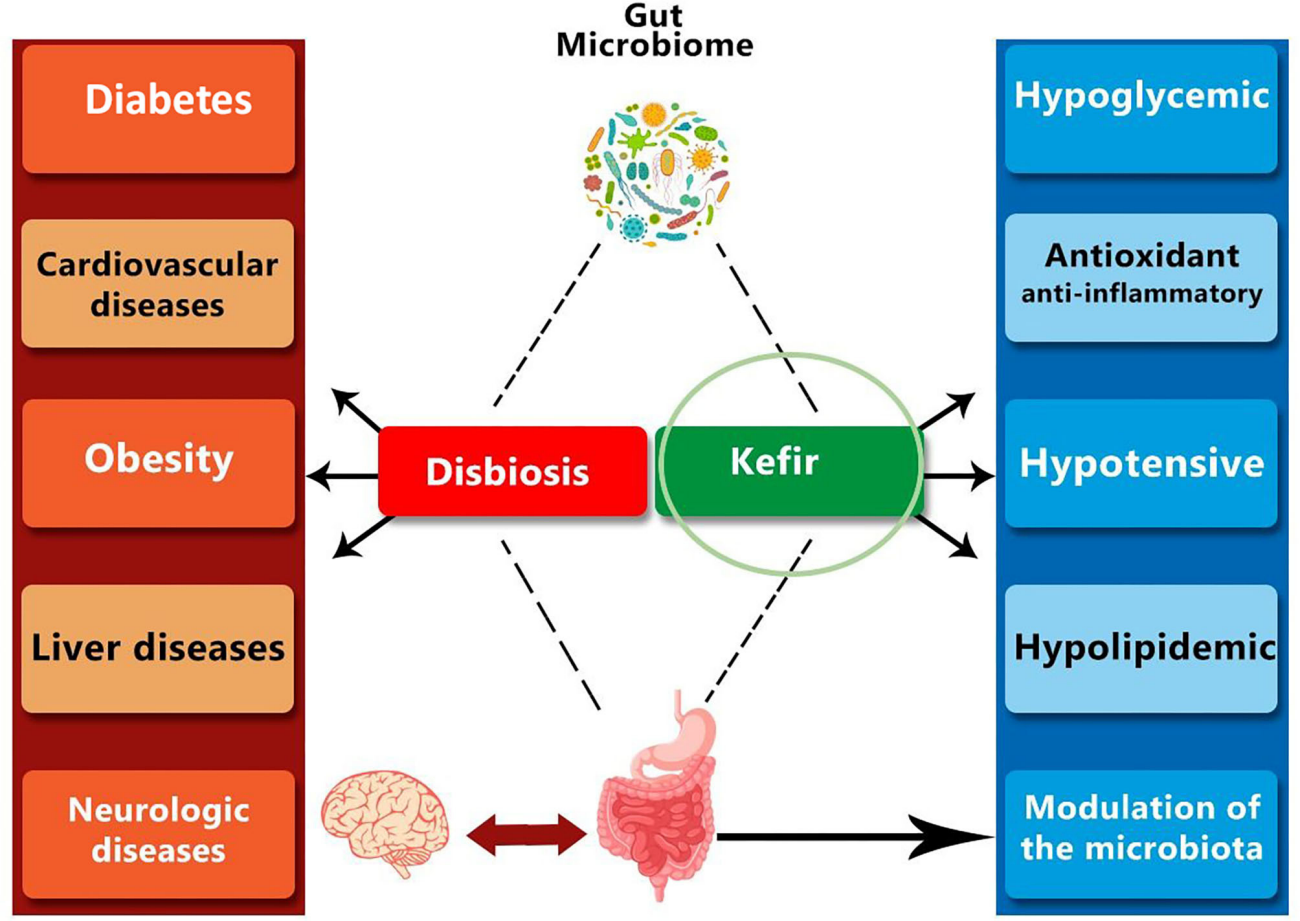
The dynamics of changes in the intestinal microbiome can lead to undesirable modifications, such as dysbiosis, triggering low-grade inflammation-related chronic diseases. On the other hand, the use of probiotics and fermented drinks, such as kefir, can have an impact on the regulation of organism homeostasis, with a direct effect on the gut-brain axis, being a possible strategy for the prevention of metabolic diseases.

Alterations in gut microbiota, in the form of dysbiosis or metabolic endotoxemia shows systemic activity (Figure 1), since they allow the occurrence of low-grade chronic inflammations that affect the organism as a whole.

The modulation of the intestinal microbiota, thus, stands out as a good strategy for the prevention and treatment of diseases. The use of fermented foods with probiotic activity is a nutritional alternative to drug treatments, and kefir, due to the absence of harmful effects regarding its consumption—in animals and humans–, low cost, ease of preparation, and microbiological composition—rich in bioactive compounds, metabolites, and peptides—stands out as a potential food with functional benefits. Besides that, promising effects as immunomodulatory, hypocholesterolemic, antihypertensive and glycemic control are expected. However, it is mandatory to deepen into the molecular mechanisms and the microorganisms involved, and more well-controlled human intervention studies are required.

## Future Perspectives

Kefir is a low-cost fermented product that is gaining interest due to its potential impact in the prevention and treatment of non-communicable diseases. Yet, the microbiological composition of kefir varies according to the geographical location, fermentation matrix (water solution with sugar, whole cow's milk, skimmed cow's milk, goat's milk, donkey milk, among others), environmental conditions (temperature and fermentation time), and grain (g)/drink (mL) ratio used in the fermentation of the product. Also, the presence of yeasts and their proportion in the drink, as well as the production conditions, among others, can generate drinks with different compositions and characteristics (Table 2). Consequently, as a future perspective, it is believed that the next studies will focus on the development of a unified production protocol, as well as on the determination of which microorganisms should be present in the starter culture and the drink, in order to consider that the final product, in fact, can be classified as a kefir.

**Table 2:** Microbiological and nutritional characterization of the kefir used in the different studies and production protocols (when available).

**References**	**Concentration**	**Microbiological and nutritional composition**	**Production protocol**
Bellikci-Koyu et al. (19)	–	- Culture DC1500I; - *Lactococcus lactis* subsp. *lactis, L. lactis* subsp. *cremoris, L. lactis* subsp. *diacetylactis, Leuconostoc mesenteroides* subsp. *cremoris, Lactobacillus kefiri, Kluyveromyces marxianus, Saccharomyces unisporus*.	- Produced in whole milk 3.5%; - Distribution to volunteers twice a week.
Bourrie et al. (28)	1% (w/v)	For commercial kefir: - *Streptococcus thermophilus, Lactobacillus delbrueckii subsp. bulgaricus, L. casei, L. acidophilus, L. delbrueckii subsp. lactis, L. rhamnosus, Bifidobacterium lactis, Lactococcus lactis* subsp. *lactis* biovar *diacetylactis, L. lactis* subsp. *cremoris, Leuconostoc mesenteroides* subsp. *cremoris*; - 8.0 × 10^6^ CFU/ml. For traditional kefir: - No information.	- Produced daily in cow's milk 2%; - Fermentation at 22°C for 18 h.
Chen et al. (29)	–	- *Lactobacillus mali* APS1; - 10^7^ to 10^10^ CFU/mg.	- Fermentation at 30°C for 12 h.
Fathi et al. (23)	–	–	- Produced by Fars Pegah Dairy Co., Shiraz, Iran.
Golli-Bennour (31)	–	- Traditional Tunisian culture, containing lactic acid bacteria; - Predominant population are lactic acid bacteria (9.5 ± 0.15 × 10^10^ CFU/g) and yeast (9.2 ± 0.14 × 10^6^ CFU/g).	- Daily production.
Kim et al. (32)	10%	- Each mL contains: 9.62 ± 0.19 Log CFU of lactic acid bacteria, 9.52 ± 0.12 Log CFU of acetic acid bacteria and 7.67 ± 0.30 Log CFU of yeast.	- Fermentation at 25°C for 24 h.
Kim et al. (33)	5% (w/v)	- *Lactobacillus kefiranofaciens, L. kefiri, L. lactis, Leuconostoc mesenteroides*.	- Fermentation at 25°C for 24 h.
Kim et al. (20)	10%	- Each mL contains: 9.32 ± 0.23 Log CFU of lactic acid bacteria and 7.12 ± 0.36 Log CFU of yeast.	- Fermentation at 25°C for 24 h.
Lim et al. (35)	1:10 (w/w)	- Grains from the KU Center for Food Safety, College of Veterinary Medicine, Konkuk University.	- Produced in UHT milk; - Fermentation ate 30°C overnight; - Production for 6 continuous weeks followed by lyophilization and storage at - 20°C.
Noori et al. (36)	–	- pH: 4.8	- Cow's milk UHT or soy milk; - Fermentation at 25°C for 24 h followed by storage at 4°C to interrupt the process.
Ostadrahimi et al. (24)	–	- *Streptococcus thermophiles, Lactobacillus casei, L. acidophilus, Bifidobacterium lactis*; *-* Fat: 0.3%.	- Weekly production.
Ozcan et al. (25)	–	- Kefir produced by Altinkiliç Company, Turquia; - No sugar and no flavor.	- Stored at 4°C and delivered to volunteers weekly.
Praznikar et al. (26)	–	- *Lactobacillus parakefiri, L. kefiri, L. kefiranofaciens* ssp. *kefirgranum, Kluyveromyces marxianus, Kazachstania exigua, Rhodosporidium kratochvilovae;* - 80% water; - pH 4.03.	- Produced by Ljubljanske mlekarne (Ljubljana, Slovenia)
Rosa et al. (37)	5% (w/w)	- Lactic acid bacteria: 2.78 × 10^7^ CFU/ml; - Yeast: 2.94 × 10^8^ cell/ml; - Lactic acid 0.806 ± 0.04 g/100 g; - Fat: 3.03 ± 0.16 g/100 g; - Protein: 3.03 ± 0.01 g/100 g; - pH 4.10 ± 0.10.	- Produced in pasteurized milk (3.5% protein, 5% carbohydrate and 3% fat); - Fermentation at 25–28°C for 24 h.
St-Onge et al. (27)	–	–	- Produced by Liberty Co, Candiac, Québec.
Sun et al. (38)	–	- Culture Collection Center of the Institute of Microbiology, Chinese Academy of Sciences (accession number CGMCC2809); - *Lactobacillus kefiranofaciens* ZW3.	–
Talib et al. (43)	10% (w/v)	- Mainly: *Lactobacillus harbinensis, L. paracasei, L. plantarum*; - All: *L. harbinensis* B22, HBUAS5305, NBRC 100982, FQ003; *L. brevis* HDRS2; *L. sp* MS6; *L. plantarum* Gt2, ZDY36a, HBUAS52249, NWAFU1558, Akhavan-Q3, Y-2-9, MSD1-4, DSR M2, LQ80; *L. paracasei* HBUAS52231, HBUAS53273; *L. casei* YQ116, H19.9.	- Produced in water solution with brown sugar; - Fermentation at room temperature for 24 h.
Tiss et al. (39)	5% (w/v)	–	- Soy milk; - Fermentation at 24 h; - Storage at 4°C (Produced every 7 days before consumption).
Tung et al. (40)	10% (w/v)	–	- Fermentation at 20°C for 20 h; - Kefir undergoes two fermentation processes: First at 5% w/v concentration and then at 10% w/v concentration.
Vinderola et al. (41)	–	–	- Produced in pasteurized milk with 1.8% fat by Les Produits de - Marque Liberté (Candiac, Québec, Canada)
Wouw et al. (42)	2% (w/v)	–	- Produced in whole milk from Irish cows; - Fermentation at 25°C for 24 h.

*Where: CFU, colony forming unit; g, gram; mg, milligram; mL, milliliters; pH, hydrogen potential; UHT, ultra-high temperature; v, volume; w, weight*.

## Study Limitations

As previously discussed, there are many factors involved in the production and dosage of kefir. This variability hinders the reproducibility of the results discussed in this narrative review and impairs future meta-analyses, considering this factor as a limitation.

## Author Contributions

MCGP has been responsible for conception of this article. MCGP and MMD have been involved in aquisition, analysis and interpretation of data, as well as in drafting the manuscript, and revising it critically. JAM and FIM have revised the manuscript critically, giving important intelectual content. All authors have read and approved the final manuscript.

## Conflict of Interest

The authors declare that the research was conducted in the absence of any commercial or financial relationships that could be construed as a potential conflict of interest.

## Abbreviations

AST: aspartate aminotransferase
ALT: alanine aminotransferase
CPT1: carnitine palmitoyltransferase 1
ELANS: American study of nutrition and health
EPM: elevated plus maze
FAS: fatty acid sintase enzyme
FST: forced swim test
GABA: gamma aminobutyric acid
GLP-1: glucagon-like peptide
HOMA-IR: insulin resistance index
IFN-γ: interferon-gama
IL-1β: interleukin-1 beta
mL: mililiter
OFT: open field test
p-AMPK: AMP-activated protein kinase
PPAR α: peroxisome proliferator-activated receptor alpha
TGF-β: transforming growth factor beta
TMAO: trimethylamine N-oxide
TMF: transplantation of fecal microbiota
TNF-α: tumor necrosis factor alpha.
